# Comparative Study of TPGS and Soluplus Polymeric Micelles Embedded in Poloxamer 407 In Situ Gels for Intranasal Administration

**DOI:** 10.3390/gels10080521

**Published:** 2024-08-09

**Authors:** Bence Sipos, Frézia Földes, Mária Budai-Szűcs, Gábor Katona, Ildikó Csóka

**Affiliations:** Institute of Pharmaceutical Technology and Regulatory Affairs, Faculty of Pharmacy, University of Szeged, Eötvös Street 6, H-6720 Szeged, Hungary; ffreesia118@gmail.com (F.F.); budai-szucs.maria@szte.hu (M.B.-S.); katona.gabor@szte.hu (G.K.); csoka.ildiko@szte.hu (I.C.)

**Keywords:** polymeric micelle, intranasal, TPGS, Soluplus, in situ gel, Poloxamer 407

## Abstract

This study aims to highlight the importance of choosing the appropriate co-polymer or co-polymer mixed combinations in order to design value-added nasal dosage forms. Local therapy of upper respiratory tract-related infections, such as nasal rhinosinusitis is of paramount importance, thus advanced local therapeutic options are required. Dexamethasone was encapsulated into three different polymeric micelle formulations: Soluplus or TPGS-only and their mixed combinations. Dynamic light scattering measurements proved that the particles have a micelle size less than 100 nm in monodisperse distribution, with high encapsulation efficiency above 80% and an at least 7-fold water solubility increase. Tobramycin, as an antimicrobial agent, was co-formulated into the in situ gelling systems which were optimized based on gelation time and gelation temperature. The sol–gel transition takes place between 32–35 °C, which is optimally below the temperature of the nasal cavity in a quick manner below 5 min, a suitable strategic criterion against the mucociliary clearance. In vitro drug release and permeability studies confirmed a rapid kinetics in the case of the encapsulated dexamethasone accompanied with a sustained release of tobramycin, as the hydrophilic drug.

## 1. Introduction

The formulation and testing of novel advanced therapeutic systems in the local treatment of nasal infections are one of the current hot trends in the pharma research and development sector. Maximizing efficacy and minimizing systemic side effects are also of paramount importance. Hence, systems which can sufficiently improve drug retention while also improving absorption in the nasal cavities are required [1,2,3]. Local treatment of nasal rhinosinusitis is generally executed via steroidal nasal sprays associated with oral antibiotics [4,5]. However, antibiotics can cause a lot of unnecessary side effects. Also, their excessive utilization can lead to antibiotic resistance [6]. Clinical practice dictates the usage of nasal steroidal anti-inflammatory drugs, but clinical research has also highlighted great results with local administration of antibiotics, such as tobramycin. As studies performed on patients with chronic rhinosinusitis via nebulization revealed, a significant improvement in symptoms and a reduction in bacterial load was achieved due to high local concentration of the drug directly at the infection site [7,8,9]. 

Tobramycin (TB) is a water-soluble drug capable of absorption in soluble form, however, most steroidal anti-inflammatory drugs, such as dexamethasone (DXM) lack this property. To enhance the water solubility, several solubilization methods can be exploited. However, current pharma R&D also highlights the advantageous utilization of nanocarriers, such as polymeric micelles [10,11]. Polymeric micelles are built by amphiphilic (graft) co-polymers. Similar to classic surfactants, they can self-assemble into associated colloidal systems. Their main advantage lies in their high solubilization capacity and higher stability in the biological milieu. Polymers are usually combined in mixed micellar systems if multiple advantages are to be exploited [12,13,14]. Many co-polymers have slighter solubilization capacity, however, they can enhance permeability better than classic solubilizers and vice versa. TPGS (D-α-tocopheryl polyethylene glycol 1000 succinate) is a derivative of vitamin E, where the enhanced solubility is due to the PEG segment of the molecule. Their average micelle size is between 10 to 100 nm based on the polymerization degree and if the polymer has been modified. It is also biocompatible and safe for use in humans. It is also known to inhibit P-gylcoprotein, improving cell permeability for active substances [15,16,17]. Soluplus (SP) is a graft copolymer composed of polyvinyl caprolactam (PVC), polyvinyl acetate (PVA), and polyethylene glycol (PEG). Besides the utilization of this excipient for its solubilization in amorphous solid dispersions, in liquid media, it forms polymeric micelles with a micelle size of approximately 80 nm. It has been proven to be a versatile excipient for passive targeting and enhances passive diffusion through various administration routes [18,19,20]. Both mentioned co-polymers are non-ionic which also reduces the risk of undesirable interactions with biological components, such as proteins in e.g. the bloodstream. They also show a minimal cytotoxic effect and do not interact with cell membranes to cause lysis, thus a safe administration can be exploited [15,19].

In our previous research work, we designed a mixed micellar composition of these co-polymers encapsulating DXM for intranasal administration [21]. While the results showed that a rapid drug release system can be achieved, questions still arose on whether their monocomponent application might hold the benefits as well. Also, a critical point in nasal drug administration is the residence time, which is typically below 20 min due to the rapid mucociliary clearance. Polymeric micelles can be utilized for quick absorption through the high surface area of the nasal cavity. However, local treatment still requires higher permeation but rather in the sustained matter. To treat the symptoms of nasal bacterial infections, two criteria are usually advised: a steroidal agent must quickly relieve the inflammatory symptoms of the disease; and the oral antimicrobial treatment should be sustained for effective treatment. The nanocarriers can provide enhanced and rapid drug release, however, this is not always the case for pure hydrophilic drugs. To further prolong the residence time for the antimicrobial agent, smart drug delivery systems can be exploited [1,22,23,24].

Poloxamer 407 is a polymer known for its versatile application. While in low concentrations, it has a solubilizing effect, and at higher concentrations, it is capable of forming in situ thermosensitive gels. Their mechanism of action is based on a sol–gel transition, where the patient would administer the sol state liquid via a nasal spray to the nasal cavity. At the temperature of the body, it would form a gel [25,26,27]. This biocompatible polymer forms a soft transparent gel suitable for topical administration due to its non-ionic and non-irritant nature [28]. Our study aims to develop advanced drug delivery systems for intranasal administration, where the quick release and permeation profiled polymeric micelles would be incorporated into Poloxamer 407 in situ gels co-formulated with tobramycin to ensure the sustained release of this hydrophilic active substance. The addition of polymeric micelle-forming co-polymers influenced the gelation tendencies of Poloxamer 407. Thus our aim was also to find the optimal gelling polymer concentration. At last, in vitro drug release and passive diffusion studies were employed to prove that a suitable nasal dosage form was developed for effective treatment of local nasal infectious diseases.

## 2. Results and Discussion

### 2.1. Finding the Optimal Polymer Concentrations via Phase Solubility Study

**Figure 1:**
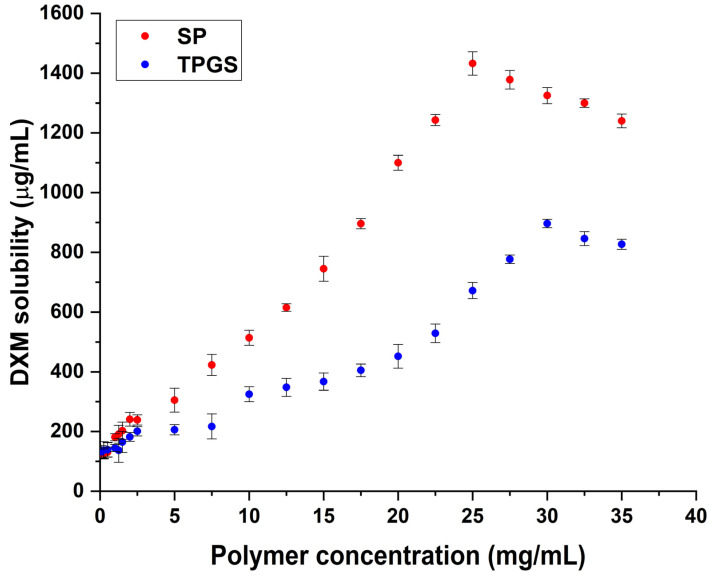
Phase solubility study of DXM in the presence of varying concentrations of Soluplus (red) and TPGS (blue) measured at 25 °C. Results are average ± SD (*n* = 3).

In this study, three formulations were proposed for comparative investigation. A binary polymeric micelle was previously reported by our research group; thus, it was used in this study as the binary formulation (DT_SP/TPGS) [21]. To investigate the effect of Soluplus (SP) and tocophersolan (TPGS) alone, a phase solubility study was performed to determine the highest solubilization efficiency at various concentrations of the polymers (Figure 1). In the case of SP, the sample with the concentration of 25 mg/mL showed the highest solubility compared to others (DT_SP). Above this, the solubility curve broke down which can be due to aggregation via the excessive amounts of polymers. A similar tendency was achieved in the case of TPGS as well, however, a higher concentration of 30 mg/mL TPGS is required in this formulation (DT_TPGS). 

### 2.2. Micelle Size, Micelle Size Distribution, and Zeta Potential Measurements

The micelle size (D_H_) and its distribution (PdI) were characterized via dynamic light scattering (DLS) after the formulation of the optimal polymeric concentration based on the phase solubility study accompanied by DLS. In the case of the TPGS micellar system (DT_TPGS), rather small particles were formed (21.43 ± 7.26 nm), which represents the lower region of the general size range of polymeric micelles (10 to 200 nm). In the case of the SP micelles (DT_SP), higher sized particles were formed (72.41 ± 6.32 nm). This corresponds to the blank polymeric sizes as well, which are 10–30 nm in the case of TPGS, and approx. 80 nm in the case of SP. It can vary based on association number and molecular weight, and naturally the general influencing factors such as temperature, pH, etc. The binary system (DT_SP/TPGS) had a slightly higher micellar size of 92.3 ± 3.4 nm. This increase can be explained by the fact that the co-polymers can form secondary linkages among themselves, and binary polymeric micelles would form in part or completely alternating around the hydrophobic core region. The PdI values of all formulations were below 0.300, whose dimensionless number corresponds to the monodisperse distribution of the polymeric micelles. Monodisperse distribution is of paramount importance in providing uniform drug release and permeation [29].

The zeta potential reflects on the surface charge at the solid–liquid surface of the nanoparticles. The lowest absolute value was in the case of DT_TPGS (−13.4 ± 2.3 mV), while the highest value was observed in the binary system, DT_SP/TPGS (−21.2 ± 2.8 mV). The higher the absolute value, the more efficient colloidal stability would be expected due to the prevention of aggregation via electrostatic repulsions. The zeta potential (ζ) also influences the absorption profile through the intranasal administration route. Negatively charged particles tend to permeate paracellular transport via passive diffusion, rather than a liberation process followed by nano-sized active substance particle absorption [30]. The results of the dynamic light scattering measurement are summarized in Table 1.

### 2.3. Determination of Encapsulation Efficiency and Thermodynamic Solubility

Regarding the encapsulation efficiency (EE) values, it can be only interpreted to DXM, since the hydrophilic tobramycin would not be encapsulated in this co-formulation. The EE values were above 80% in all cases as seen in Table 2, and the highest value was gained at the binary polymeric micelle system. This proves the superiority of the utilization of multiple co-polymers regarding solubilization. This additive value can be experienced in various mixed micellar systems in the literature as well, where the advantageous solubilization can be paired with permeability enhancement. TPGS as mentioned before is a classic solubilizer, however, its solubilization capacity is not as efficient as its permeability enhancement function [31,32,33]. SP on the other hand can be interpreted the other way around, with high solubilization and moderate permeability enhancement. The results of the EE determination correlate with the increase in thermodynamic solubility. The higher the EE, the higher the thermodynamic solubility values were. An 11-, 7-, and 13-fold solubility increase was experienced via the utilization of SP, TPGS, and the binary combination, respectively. The results are summarized in Table 2.

### 2.4. Finding the Optimal In Situ Gel-Forming Polymer Concentration via the Determination of Gelation Time and Temperature 

**Figure 2:**
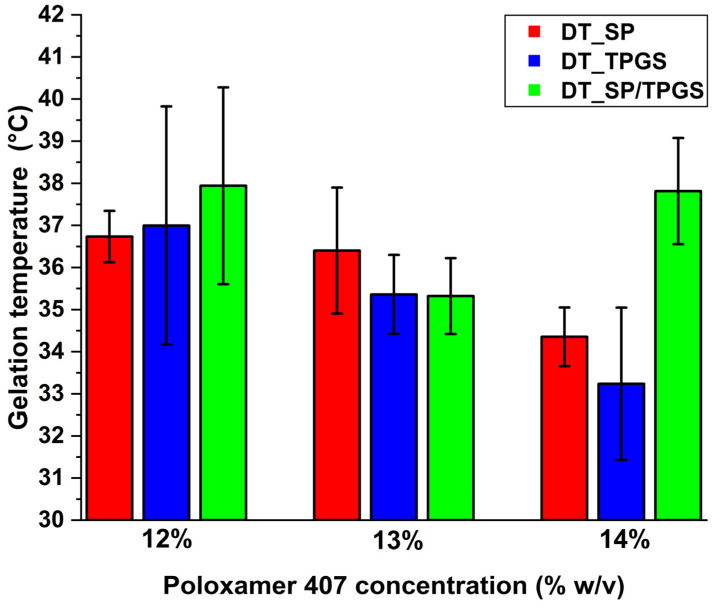
Gelation temperature measured via rotational viscosimetry at various Poloxamer 407 concentrations. DT_SP formulation: red, DT_TPGS formulation: blue, DT_SP/TPGS formulation: green. Results are average ± SD (*n* = 3).

Gelation temperature and time were measured during the optimization of the concentration of the in situ gel-forming material, i.e., Poloxamer 407. Rotational viscosimetric measurements were performed to detect the point where the sol-gel transformation took place. Based on these measurements, it can be claimed that 14% *w*/*v* of Poloxamer 407 is required for the mono systems, i.e., where only SP or TPGS is found. Regarding the mixed micellar system (DT_SP/TPGS), an increase in gelation temperature was found at higher polymer concentrations. This could be either explained by the fact that Poloxamer 407 can also form micellar structures and a ternary mixed micelle would be formed, as a classic micelle or combined with other polymeric and non-polymeric components. This is supported in the literature as well, where all the polymers are capable of being a part of a ternary mixed micelle. This phenomenon directly affects gelation properties; therefore, 13% *w*/*v* was utilized in the case of DT_SP/TPGS [34,35,36]. The gelation temperatures meet the requirements of a nasal dosage form since the temperature of the nasal cavity can be set between 32 and 35 °C, thus the sol-gel transformation slightly before this temperature is optimal (Figure 2) [37]. The other criterion is that the gelation temperature should be way higher than the ambient temperature, so a minimum temperature of 32 °C was also appointed.

**Figure 3:**
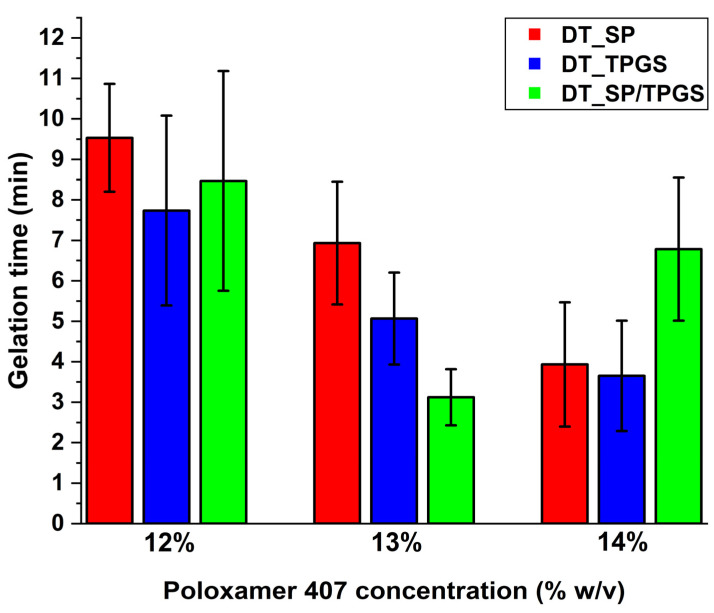
Gelation time measured via rotational viscosimetry at various Poloxamer 407 concentrations. DT_SP formulation: red, DT_TPGS formulation: blue, DT_SP/TPGS formulation: green. Results are average ± SD (*n* = 3).

The gelation time also met the requirements of a general nasal dosage form. The average residence time in the nasal cavity can be placed between 10 to 15 min, where the mucociliary clearance can eliminate the administered materials with a speed of 1–2 mm/h in the inferior region and 8–10 mm/h in the posterior region [38]. Thus, rapid gelation is favorable, since the formulation strategy requires working against the mucociliary clearance. The quickest gelation occurred at 13% *w*/*v* in the case of the binary system, while similar tendencies were found at 14% *w*/*v* Poloxamer concentration in the case of only SP or TPGS-containing other formulations (Figure 3).

### 2.5. Characterization of the In Situ Gel Formulations

The pH of the nasal cavity ranges in the slightly acidic pH range between 5.5 to 6.5, which is influenced by various conditions such as health-related factors, acute or chronic infections, etc. Generally, the recommended pH of a nasal dosage form is between 5.0 and 7.4, which would not cause any irritations, adverse effects, or irregular drug release and absorption behavior [39,40]. In all cases, the pH met the requirements in sol and gel states (Table 3). The transparency of the formulations was also investigated under light before a white and black background. A soft, transparent gel can be visually observed in all cases. The drug content, as the amount of recovered active substance from the initially measured materials, is also above 90%.

### 2.6. In Vitro Mucoadhesion Study

**Figure 4:**
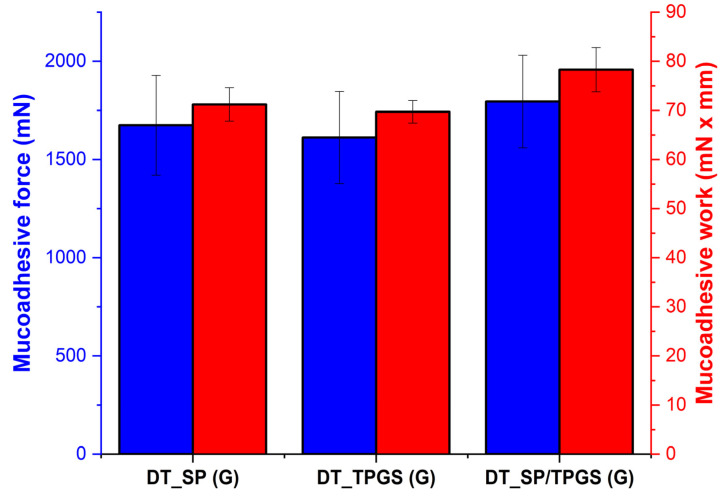
In vitro mucoadhesion study—mucoadhesive force (blue) and mucoadhesive work (red) regarding the different in situ gelling formulations showing a moderately high mucoadhesive tendency. Results are average ± SD (*n* = 5).

Poloxamer 407 is known for its gelling properties. Besides the physical prolonging of the residence time, it has mucoadhesive attributes. It can form hydrogen bonds with the mucin glycoproteins present in the mucus layer, hydrophobic interactions can also occur with the hydrophobic domains within the mucus and the swelling mechanism will lead to an entanglement within the mucin network, providing anchorage-enhancing adhesion [36,41]. No significant difference was observed among the investigated formulations, the only difference is based on the varying concentration of the application (Figure 4). These moderately high mucoadhesive force and work values also represent adequate adhesive properties suitable to work against mucociliary clearance. In comparison, Jones et al. investigated the viscoelastic and mucoadhesive properties where higher adhesive force values were found when the Poloxamer 407 was mixed with Carbopol 934P in a binary polymeric system. However, the sol–gel transition temperature also decreased significantly at the highest mucoadhesive values [42]. Therefore, these moderately high values obtained in the formulations are not only suitable for prolonging the residence time in the nasal cavity, but also makes it possible to be able to administer this formulation out of a nasal spray or a drop. 

### 2.7. In Vitro Drug Release Study

**Figure 5:**
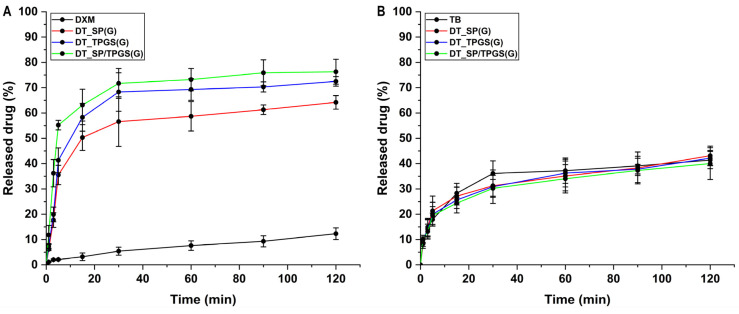
In vitro drug release profile of dexamethasone (**A**) and tobramycin (**B**) from the formulations and their references showing enhanced drug release in the case of the dexamethasone-loaded polymeric micelles. Results are average ± SD (*n* = 3).

Based on the in vitro drug release curves, the release of DXM increased from the polymeric micellar formulations in all cases. It shows a burst-like drug release profile despite the fact that a drug release hindering polymer was applied as a matrix. A slight difference in the case of the binary system and the DT_TPGS formulation is experienced but only the binary system is significantly higher compared to the DT_SP formulation (*, *p* < 0.05) after 15 min. Compared to the reference DXM suspension, all formulations are significantly higher (***, *p* < 0.001). Regarding the drug release of tobramycin, no significant difference can be observed compared to the initial TB. In the case of this hydrophilic drug, a prolonged release profile was achieved (Figure 5).

### 2.8. In Vitro Nasal Passive Diffusion Study

**Figure 6:**
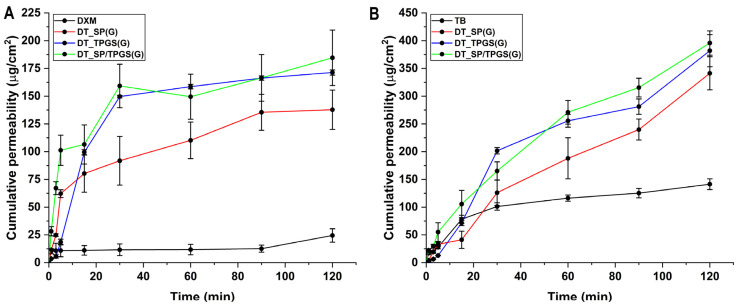
In vitro nasal passive diffusion study of dexamethasone (**A**) and tobramycin (**B**) from the formulations and their references. Results show the enhanced permeation profile of the dexamethasone-loaded polymeric micelles and in the case of tobramycin. Results are average ± SD (*n* = 3).

As a result of the in vitro nasal passive diffusion study, it can be seen that in all cases, a higher degree of permeation was achieved compared to the reference DXM. The difference between the three polymer systems is striking: the penetration enhancer TPGS takes on higher values compared to the SP-containing system, with the binary system having the highest value (Figure 6). Tobramycin had a drug release percentage up to approximately 40% in this 2-h period. This might be due to the fact that no functionalization, structure change, encapsulation, etc., was performed on TB, which would not lead to an enhanced drug release rate compared to DXM in the polymeric micelle formulation. In comparison to drug release studies performed with Poloxamer 407 hydrogels, higher percentages could be achieved with other administration routes where the achievable residence time is found to be significantly higher, i.e., peroral, subcutan, etc., administration. However, due to the limitations of the nasal cavity’s mucociliary clearance, an increased residence time of 1 to 2 h can be considered good. As the study was performed in vitro, it is a must to mention that other factors also contribute to the change in drug release, such as the erosion of the hydrogel structure [41,43,44]. 

The drug permeation curves reflect on the calculated permeability-related parameters as well (Table 4), where a similar trend can be observed. Flux (J) is a unit of concentration that indicates how much active substance has passed through a given diffusion matrix. In the case of the flux, the last point (the absolute flux) can be compared where the same tendency of increase in the case of DT_TPGS and DT_SP/TPGS can be seen. The permeability coefficient and the apparent permeability also follow this trend. The results also contribute to the advantageous utilization of SP and TPGS in a mixed micelle system. Similarly, Zhou et al. developed paliperidone-loaded mixed micelles also providing enhanced drug permeation, which was corroborated by in vivo results [33]. 

## 3. Conclusions

In conclusion, it can be claimed that optimization based on solubility enhancement properties of various polymeric micelle-forming co-polymers is crucial and the benefit must be further evaluated via thorough, quality-driven investigations. This research is significant as it offers potential improvements in the enhancement of bioavailability in the nasal cavity with a direct approach to treating local bacterial infections. Improved patient compliance might also be expected regarding the application of these types of systems, where quick relief of inflammatory symptoms associated with effective antimicrobial therapy can be achieved. The enhanced drug release and measured permeability-related factors would propose a better tissue absorption of the active agents. The local administration of the active substances would also prosper the added value as it would increase patient adherence. Overall, polymeric micelles with proper colloidal properties embedded in Poloxamer 407 thermosensitive gels might be a successful alternative as a local therapeutic combination compared with current therapies of nasal steroidal sprays combined with peroral antibiotics.

## 4. Materials and Methods

### 4.1. Materials

Dexamethasone (DXM) and tobramycin (TB) as model drugs were purchased from Sigma-Aldrich Co., Ltd. (Budapest, Hungary). Soluplus (SP, poly(vinyl caprolactam)—poly(vinyl acetate)—poly(ethylene glycol) graft co-polymer (PCL-PVAc-PEG)) was kindly gifted from BASF GmbH (Hannover, Germany). TPGS (tocophersolan, D-α-tocopheryl polyethylene glycol 1000 succinate); Poloxamer 407 (poly(oxyethelene)—poly(oxypropylene)—poly (oxyethylene) (PEO-PPO-PEO); D-trehalose dihydrate (D-TRE), mucin from porcine stomach (type III), salts required for phosphate buffer saline (PBS; pH = 7.4), simulated nasal electrolyte solution (SNES)—8.77 g/L sodium chloride, 2.98 g/L potassium chloride, and 0.59 g/L calcium chloride dihydrate were also acquired from Sigma-Aldrich Co., Ltd. (Budapest, Hungary).

### 4.2. Quantitative Analysis of Dexamethasone via Liquid Chromatography

The quantitative determination of DXM was performed via high-pressure liquid chromatography (HPLC) using an Agilent 1260 Infinity (Agilent Technologies, Santa Clara, CA, USA) instrument. The stationary phase was a Gemini^®^ NX 5u C18 110 Å column (5 µm, 150 mm × 4.6 mm (Phenomenex, Torrance, CA, USA)). The mobile phases were the following: (A) purified water and (B) acetonitrile—methanol 50:50 mixture, and they were applied in a 55:45 ratio. The injection volume was 10 µL. The separation was performed by isocratic elution for 10 min at 40 °C with a flow rate of 1 mL/min. Chromato-grams were detected at 254 ± 4 nm using a UV-Vis diode array detector. The retention time was 7.57 min. The limit of detection (LOD) and limit of quantification (LOQ) of DXM were 14.46 and 43.82 ppm, respectively. The calibration was performed from 2 to 10 µg/mL and from 0.2 to 1.0 mg/mL, where the determination coefficients of linearity (R^2^) values were 0.9996 and 0.9992, respectively. Chromatograms were evaluated using ChemStation B.04.03 Software (Agilent Technologies, Santa Clara, CA, USA) [21].

### 4.3. Quantitative Analysis of Tobramycin via Liquid Chromatography

The Agilent 1260 (Agilent Technologies, Santa Clara, CA, USA) instrument was also used for the quantification of TB, where the stationary phase was a Chrom-Clone™ C18 110 Å column (diameter: 5 µm; 150 mm × 4.6 mm (Phenomenex, Torrance, CA, USA)). The injection volume was 20 µL. The mobile phases were the following: (A) 0.02 M PBS (pH = 10.0), and (B) acetonitrile in the ratio of 30:70. The isocratic separation was performed for 7 min at 40 °C. Chromatograms were detected at 215 ± 4 nm. The retention time of TB was at 3.51 min. The calibration was performed from 1 to 10 µg/mL and from 0.1 to 5.0 mg/mL, where the determination coefficients of linearity (R^2^) values were 0.9995 and 0.9997, respectively. The LOD and LOQ values were 16.54 and 51.64 ppm, respectively. Chromatograms were evaluated using ChemStation B.04.03 Software (Agilent Technologies, Santa Clara, CA, USA).

### 4.4. Phase Solubility Study of Dexamethasone

Phase solubility test was performed with varying concentrations of TPGS and SP in the concentration range of 0.01–35 mg/mL region. As a dissolving media, a 3 mg/mL solution of tobramycin in purified water was used to represent the conditions of the final formulation. A physically observable excess amount of DXM was added to each vial and they were stirred constantly for 72 h at ambient temperature (750 rpm, 25 °C). The suspensions were filtered with a 0.22 µm pore-sized polyethersulfone membrane. The clear solutions’ DXM content was measured via HPLC.

### 4.5. Formulation of Dexamethasone-Loaded Polymeric Micelles

During the development of polymeric micelles, it was aimed to contain DXM in a concentration of 0.5 mg/mL as a model concentration and it should be co-formulated with 3 mg/mL of TB. At first, 6 mg/mL TB was dissolved in purified water followed by the addition of 25 mg/mL of SP (DT_SP sample) and 30 mg/mL TPGS (DT_TPGS sample), or 12 and 8 mg/mL of SP and TPGS (DT_SP/TPGS sample), respectively. A total of 10 mL of the stock solutions were prepared, each sample containing TB and the dissolved polymers. The next step was to mix the tert-butanolic solution of DXM (1 mg/mL) in the same volume. To the resulting mixture, 5% *w*/*v* D-TRE was dissolved as a cryoprotectant and the system was left incubated and stirred for 4 h (ambient temperature, 750 rpm). Out from the mixtures, volumes of 1 mL were pipetted into a freeze-drying vial, then the samples were frozen via a Scanvan CoolSace 100-9 (LaboGene Aps, Lynge, Denmark) laboratory apparatus to −40 °C. The freeze-drying was performed at −40 °C at 0.013 mbar for 12 h. The secondary drying was performed at 25 °C at 0.013 mbar for 6 h. To characterize the products, the freeze-dried cakes were dissolved in 1 mL of purified water with resultant concentrations of 0.5 mg/mL DXM and 3 mg/mL TB. 

### 4.6. Characterization of Polymeric Micelles

#### 4.6.1. Determination of Micelle Size, Size Distribution, and Zeta Potential

The micelle size, expressed as the Z-average (average hydrodynamic diameter), and the micelle size distribution, expressed as polydispersity index (PdI) of the DXM-loaded polymeric micelles in the presence of tobramycin was measured via a Malvern Nano ZS Zetasizer (Malvern Instruments, Malvern, UK) apparatus based on dynamic light scattering. The in-water dissolved samples were measured at 25 °C in folded capillary cells with a refractive index of 1.592. The zeta potential (ζ) of the formulations was also measured. All measurements were carried out in triplicate (*n* = 3), and the results were expressed as average ± SD.

#### 4.6.2. Determination of Encapsulation Efficiency

TB is a water-soluble drug; thus, the encapsulation efficiency (EE) cannot be interpreted since it will not be incorporated into the core of a polymeric micelle. To determine the EE for DXM, the indirect method was applied. Freeze-dried cakes were dissolved in 1 mL of purified water, placed in Eppendorf tubes, and then separated from the aqueous media via centrifugation using a Hermle Z323 K high-performance refrigerated centrifuge (Hermle AG, Gosheim, Germany) at 12,000 rpm, 4 °C for 45 min. Quantitative measurements were performed with the supernatant via HPLC [21]. All measurements were carried out in triplicate (*n* = 3), and the results were expressed as average ± SD. EE was calculated via the following equation:

(1) $$EE=\frac{Initial\;DXM\;\left(mg\right)-Measured\;DXM\;(mg)}{Initial\;DXM\;(mg)}\times 100$$

#### 4.6.3. Determination of Thermodynamic Solubility

In the case of polymeric micelles, an important parameter is the degree of increase of water solubility achieved during the encapsulation process. Thus, the thermodynamic solubility was measured via the saturation method. The three formulations were dissolved separately in excess in 1 mL of purified water, then they were stirred constantly for 72 h at ambient temperature (25 °C, 750 rpm). After that, the undissolved particles were filtered through a polyethersulfone membrane filter with a pore diameter of 0.22 µm, and the concentration of the pure solutions was measured using HPLC [21]. All measurements were carried out in triplicate (*n* = 3), and the results were expressed as average ± SD.

### 4.7. Incorporation of Polymeric Micelles and Tobramycin into In Situ Gelling Systems

After the freeze-drying process, solutions of Poloxamer 407 were prepared in the concentration range of 12 to 14% *w*/*v* at 4 ± 1 °C. After complete dissolution, each freeze-dried cake was dissolved in 1 mL of the Poloxamer 407 solutions (1000 rpm, 10 °C). The storage of the sol state formulations was carried out in a refrigerator (4 ± 1 °C). The selection of the concentration range was based on preliminary research and prior experience with protein-based nanocarriers embedded in Poloxamer 407 gels [45].

### 4.8. Characterization of the In Situ Gelling Formulations

#### 4.8.1. Determination of Gelling Time and Temperature of the In Situ Gelling Formulations

To determine the sufficient gelling time and temperature, rheological measurements were carried out via a Physica MCR302 rheometer (Anton Paar, Graz, Austria). A cone and plate-type measuring device with a cone angle of 1° was applied. The cone diameter was 25 mm with a gap height of 0.046 mm. The gelation temperature was measured while the temperature was increased from 20 to 40 °C with a heating rate of 1 °C/min. The gelation time was followed at a constant frequency of 1.0 rad/min and a strain of 1% at 37 °C. The samples were measured immediately and stored at 4 ± 1 °C prior to the experiment. All measurements were carried out in triplicate (*n* = 3), and the results were expressed as average ± SD.

#### 4.8.2. Determination of pH, Clarity, and Drug Content of the In Situ Gelling Formulations

The pH of the in situ gelling formulation in sol and gel states was measured via a dipping pH meter (WTW^®^ inoLab^®^ pH 7110 laboratory pH tester, Thermo Fisher Scientific, Budapest, Hungary). The clarity of the in situ gelling formulations was also characterized in sol and gel states by visual examination against white and black backgrounds under light. The drug content of the gels was determined in the gel state, where a precise amount of the gel was measured followed by a breakup in 10 mL of methanol. The dispersion was vortexed for 10 min to break the gel structure, then filtered through a 0.45 µm pore-sized polyethersulfone (PES) membrane. The DXM and TB content were measured by HPLC. All measurements were carried out in triplicate (*n* = 3), and the results were expressed as average ± SD.

### 4.9. In Vitro Nasal Applicability Investigations

#### 4.9.1. In Vitro Mucoadhesion Study

The in vitro determination of mucoadhesion was performed via the tensile test. A TA-XT Plus texture analyzer (Metron Ltd., Budapest, Hungary) equipped with a cylinder probe with a 1 cm-in-diameter and a 5 kg weighted load cell was used. Filter paper wetted with 50 µL of artificial mucus (8% *w*/*v* mucin in SNES) was fixed in the mucoadhesion rig of the instrument. A total of 20 mg of the samples was applied for each measurement. The two surfaces were pressed against each other for 3 min with a force of 2500 mN, then the probe body was lifted from the surface with a velocity of 2.5 mm/min. The mucoadhesive force was measured, then mucoadhesive work was calculated from the adhesive force-time curves as its area under the curve (AUC) values. Five parallel measurements were performed (*n* = 5), and the results were expressed as average ± SD.

#### 4.9.2. In Vitro Drug Release Study

In vitro drug release study was performed in SNES at 37 °C using the dialysis bag method (Hanson SR8 Plus (Teledyne Hanson Research, Chatsworth, CA, USA)). A total of 1 mL of the sol state formulation and the references (in-sol dispersed DXM and TB) were placed in Spectra/Por^®^ dialysis bags (Spectrum Laboratories Inc., Rancho Dominguez, CA, USA; MWCO: 12–14 kDa) with Spectra/Por^®^ closures (Spectrum Laboratories Inc., Rancho Dominguez, CA, USA) and immersed in 50 mL of dissolution media. The test was conducted under 50 rpm constant stirring. Aliquots were withdrawn at predetermined time intervals of up to 120 min. All measurements were carried out in triplicate (*n* = 3), and the results were expressed as average ± SD. Quantification was performed via HPLC.

#### 4.9.3. In Vitro Nasal Passive Diffusion Study

To determine the passive diffusion tendencies of the formulations, a modified Side-bi-Side^®^ horizontal diffusion cell was applied. A regenerated cellulose membrane (Whatman™ (0.45 µm, 25 mm)) was impregnated with isopropyl myristate with a surface of 0.785 cm^2^ and used as the diffusion barrier between the acceptor and donor compartments. The compartments’ volumes were 9 mL and the investigation temperature was 36.5 °C. The donor phase consisted of the formulations dissolved in SNES and the acceptor phase was a pH 7.4 PBS. At predetermined time points, 50 µL aliquots were taken from the acceptor phase and the concentrations of DXM and TB were measured via HPLC. The taken aliquots were immediately replaced with the same volume of pH 7.4 PBS. Flux (J), the cumulative permeability per time, was calculated via the following equation:

(2) $$J=\frac{m_{t}}{A\times t}$$

The permeability coefficient was calculated via the following equation:

(3) $$K_{p}=\frac{J}{C_{d}}$$

And lastly, the apparent permeability coefficient was calculated via the following:

(4) $$P_{app}=\frac{∆{\left[C\right]}_{A}\times \;V_{A}}{A\times {\left[C\right]}^{D}\times ∆t}$$

In the equations, the following abbreviations were applied: m_t_—mass of API in the acceptor chamber; A—membrane surface area (0.785 cm^2^); t—time (h); C_d_—concentration of the donor compartment; ∆[C]_A_—change of concentration in the acceptor compartment at t time; V_A_—volume of acceptor chamber (9 cm^3^); [C]^D^—concentration of the donor chamber at 0 time; ∆t—elapsed time.

### 4.10. Statistical Analysis

All presented data are in means ± SD. The significance of differences was calculated via one-way ANOVA with a post hoc test (Tukey’s multiple comparisons test, α = 0.05). Changes were considered statistically significant at *p* < 0.05. Statistical analysis was performed using TIBCO Statistica^®^ 13.4 (Statsoft Hungary, Budapest, Hungary) software.

## Figures and Tables

**Table 1:** Micelle size (D_H_), micelle size distribution (PdI), and zeta potential (ζ) values of the investigated formulations with the optimal polymer concentrations. Results are average ± SD (*n* = 3).

	DT_SP	DT_TPGS	DT_SP/TPGS
D_H_ (nm)	72.41 ± 6.32	21.43 ± 7.26	92.30 ± 3.4
PdI	0.119 ± 0.004	0.169 ± 0.011	0.249 ± 0.027
ζ (mV)	−18.4 ± 4.1	−13.4 ± 2.3	−21.2 ± 2.8

**Table 2:** Encapsulation efficiency (EE) and thermodynamic solubility (S) at 25 °C of DXM after the encapsulation into the polymeric micelles. Results are average ± SD (*n* = 3).

	DXM	DT_SP	DT_TPGS	DT_SP/TPGS
EE_DXM_ (%)	-	89.13 ± 2.70	83.05 ± 3.3	93.77 ± 4.1
S_DXM,25°C_ (µg/mL)	124.13 ± 4.51	1432.21 ± 28.79	896.25 ± 32.88	1658.02 ± 22.49

**Table 3:** pH at sol and gel state, clarity, and drug content of the in situ gelling systems. The (G) after the sample names represent the gel state of the formulations. Results are average ± SD (*n* = 3).

	DT_SP (G) (13%)	DT_TPGS (G) (14%)	DT_SP/TPGS (G) (13%)
pH_sol_	6.58 ± 0.23	6.67 ± 0.13	6.71 ± 0.15
pH_gel_	6.83 ± 0.11	6.91 ± 0.20	7.02 ± 0.09
Clarity	Transparent	Transparent	Transparent
Drug content (DXM) (%)	92.13 ± 4.12	91.39 ± 3.55	97.35 ± 1.06
Drug content (TB) (%)	94.24 ± 2.31	92.11 ± 0.87	95.45 ± 2.15

**Table 4:** The calculated permeability-related parameters from the in vitro nasal passive diffusion study. Results are average ± SD (*n* = 3).

		DT_SP (13%)	DT_TPGS (14%)	DT_SP/TPGS (13%)
	DXM			
J (µg/cm^2^ × h)	24.5 ± 2.90	137.76 ± 17.72	171.3 ± 13.21	184.6 ± 25.09
K_p_ (cm/h)	0.0245	0.1378	0.1713	0.1846
P_app_ (×10^−4^ cm/s)	1.23	6.89	8.57	9.23
	TB			
J (µg/cm^2^ × h)	141.4 ± 9.6	341.3 ± 29.9	381.9 ± 28.9	395.8 ± 21.9
K_p_ (cm/h)	0.0236	0.0569	0.0634	0.0659
P_app_ (×10^−4^ cm/s)	1.18	2.84	3.18	3.30

## Data Availability

Data are available upon request from the authors.
